# Efficacy of homemade face masks against human coughs: Insights on penetration, atomization, and aerosolization of cough droplets

**DOI:** 10.1063/5.0061007

**Published:** 2021-09-14

**Authors:** Bal Krishan, Dipendra Gupta, Gautham Vadlamudi, Shubham Sharma, Dipshikha Chakravortty, Saptarshi Basu

**Affiliations:** 1 Department of Mechanical Engineering, Indian Institute of Science, Bangalore, Karnataka 560012, India; 2 Department of Microbiology and Cell Biology, Indian Institute of Science, Bangalore, Karnataka 560012, India; 3 Center of Biosystems Science and Engineering, Indian Institute of Science, Bangalore, Karnataka 560012, India; 4 Interdisciplinary Centre for Energy Research (ICER), Indian Institute of Science, Bangalore, Karnataka 560012, India

## Abstract

Ever since the emergence of the ongoing COVID-19 pandemic, the usage of makeshift facemasks is generally advised by policymakers as a possible substitute for commercially available surgical or N95 face masks. Although such endorsements could be economical and easily accessible in various low per-capita countries, the experimental evidence on the effectiveness of such recommendations is still lacking. In this regard, we carried out a detailed experimental investigation to study the fate of a large-sized surrogate cough droplet impingement at different velocities (corresponding to mild to severe coughs) on various locally procured cloth fabrics. Observation shows that larger ejected droplets (droplets that would normally settle as fomites in general) during a coughing event have enough momentum to penetrate single-layer cloth masks; the penetrated volume atomize into smaller daughter droplets that fall within aerosol range, thereby increasing infection potential. Theoretically, two essential criteria based on the balances of viscous dissipation-kinetic energy and surface tension-kinetic energy effects have been suggested for the droplet penetration through mask layers. Furthermore, a new parameter called *η* (the number density of pores for a fabric) is developed to characterize the volume penetration potential and subsequent daughter droplet size. Finally, the effect of mask washing frequency is analyzed. The outcomes from the current study can be used as a guide in selecting cloth fabrics for stitching multi-layered.

## INTRODUCTION

The current pandemic has drastically impacted the lives of people from all walks of life across the globe. Severe acute respiratory syndrome-associated coronavirus (SARS-CoV-2) is spread through pathogen-loaded respiratory droplets.^1,2^ The transmission potential of SARS-CoV-2 through the virus-loaded respiratory droplets ejected during coughing is drastically higher^3,4^ compared to other respiratory infections. Environmental droplet contamination is caused when these virus-loaded respiratory droplets are ejected into the atmosphere through breathing, speaking, singing, coughing, or sneezing by an infected person. Larger droplets tend to settle as fomites;^5,6^ however, the primary concern lies with the smaller aerosolized droplets that remain suspended in air and can be inhaled by healthy individuals. The infection probability depends on the initial viral load and other factors like viral endurance in different ambient conditions like temperature and relative humidity. A statistical meta-analysis done by various research groups worldwide has recommended wearing face masks as a control measure against both symptomatic and asymptomatic spreads.^7–11^ Approximately 100 nm in diameter, COVID-19 is a positive-strand RNA virus and makes its way from the nose to lungs. The virus spike protein attaches to the ACE2 receptor, following which the virus particle is endocytosed into the cells, and the infection cycle starts. The first line of defense that remains for us is using a facemask.

The masks act as a physical barrier to respiratory droplets, but the effectiveness of cloth-mask depends on the design, texture, and textile material used.^12^ Human simulators are used to check how far the droplets travel with and without different types of masks while speaking and coughing. All types of masks have been effective in reducing the environmental droplet contamination range around a source.^13,14^ Another experimental investigation has been carried out by Rodriguez-Palacios *et al.*,^15^ with a horizontal spray of bacteria-laden droplets where they studied the deposition concentration at different distances from the spray with and without a textile barrier. Different research groups have investigated the filtration efficiency of a surgical mask, procedure mask, and different types of cloth masks. Based on observations, the double layer mask when used with a better fit has a comparatively higher filtration efficiency.^16–20^ Mask studies can be ideally incorporated in the available multiscale physics-based disease spread model to estimate the probability of infection.^21^

Fluid dynamically droplet impact on a facemask is similar to impingement on porous networks like metallic wire meshes,^19–25^ fibers,^26–30^ and textiles.^31^ Sharma *et al.*^32^ focused on the effect of the number of layers present in a surgical mask and showed that the number of cough droplets penetrating through the mask is significantly reduced with each additional mask layer. The experiments were performed for droplet sizes of ∼300–600 *μ*m and velocity range of 2–10 m/s (Ref. 32) mimicking real cough events. The penetration of the impacting droplet depends on the surface wettability conditions,^20,23,33,34^ mesh sizes,^23,24^ fluid properties,^28^ and impact velocities^23,27^ of the droplets. At higher impingement velocities above a critical impact limit, the impinging droplet will always penetrate through the fiber pores irrespective of the wettability conditions of the fabric.^28^ The fragmentation of a water droplet impinging on mesh results in polydisperse droplets,^23^ and a critical Reynolds number exists for the penetration of droplets to occur.^35^

The outbreak of COVID-19 caused by SARS-CoV-2 is at its worst globally, especially in the low- and mid-income countries. Almost 800 000 positive cases are reported daily throughout the world, out of which about 360 000 cases correspond to India alone, making it the worst-hit country (WHO, 29th, April 2021). The WHO recommended N95 masks cost around ₹95–₹165 even after the government of India subsidy.^36^ Despite this, the cost of the N95 mask is not affordable for the lower-income population of the country with an average daily income of ₹355.^37^ Also even though the surgical masks are less expensive (₹10/piece), they need to be bought recurrently (disposable), making them unaffordable for underprivileged groups. As a feasible solution, the usage of homemade facemask (cotton mask) or face covering (handkerchief, towel) is generally recommended by policymakers;^38^ however, the quantification of the most suitable fabric is still lacking. These substitutes are popular among such populations due to breathability and convenience (as they are washable and reusable) but are not tested for penetration/aerosolization characteristics, which is done in the present study.

**FIG. 1.**
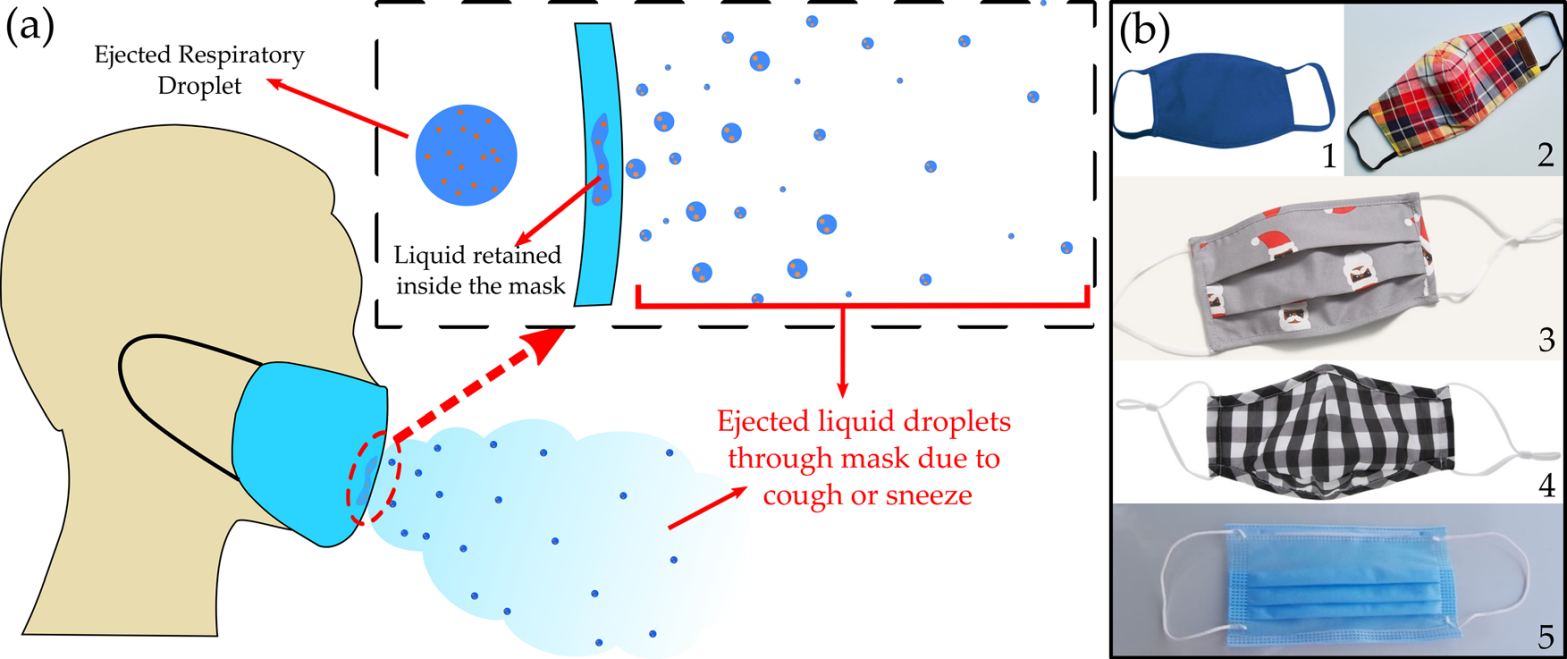
Mechanism of droplet atomization through various cotton face masks: (a) a schematic of droplet penetration and breakup through a face mask during a sneeze/cough. (b) (1)–(4): Commercially available or homemade cloth-mask, (5): surgical-mask (SM).

Due to the above reasons, many people are unable to use commercially available N95 and surgical masks and are bound to use cloth masks or makeshift masks (such as a handkerchief, towel, t-shirts) (Fig. 1). These materials are not designed for masking purposes, and the aerosol-blocking/generation efficiency is not well understood, leading to a false sense of protection. This warrants a detailed study on the effectiveness of such material against aerosol dispersion. A single-layered mask efficacy is lower than a multi-layer mask and has been investigated in the existing literature.^32^ However, this remains a secondary focus of our work. We primarily focus on identifying the most suitable and easily accessible fabric for making a homemade face mask, giving a framework for manufacturer/user to select a suitable material. Since this is a fundamental study, it was preferable to investigate the effectiveness of a single layer mask and get insight into droplet penetration/atomization for different types of fabrics. The most suitable fabric can then be used as a multi-layer mask.

The existing experimental studies that were conducted on the efficacy of cloth masks were primarily observation based. Even though recommendations were advocated, no parameter was proposed to determine the effectiveness of the fabric in blocking the droplets. In the current study, we present evidence-based recommendations on the usage of such ordinarily available fabric materials. Furthermore, we provide insight into the physical mechanism of filtration by a mask, showing that how the fabric properties like porosity and pore size influence the extrusion of impinging model cough-droplets through the fabric. The subsequent daughter droplet size distribution, after passing through the mask, is essential to determine the lifetime of these aerosolized droplets and the extent of infection. The number count of large-sized cough droplets (≥550 mm) is small. However, a significantly large percentage of the total volume is carried by these droplets as given by Duguid (1946)^39^ (also replotted in the supplementary material, see Fig. S1) where this volume was 72.22% for droplet sizes ≥600 mm and 94.21% for droplet sizes ≥250 mm. Duguid (1946)^39^ have shown a direct correlation between pathogens amount with droplet volume; therefore, the atomization of such large size droplets is relevant for disease transmission. Furthermore, such large droplets (≥550 mm) will deposit on the ground when ejected in the absence of a facemask and will have a very short airborne lifetime.^40,41^ However, if the mask is worn by an infected person, though it blocks a significant fraction of the droplet volume, it causes secondary atomization through the small pores of the mask fabric.^32^ The daughter droplets, thus, formed (typically of size ≤100 μm) fall within the aerosol range. Therefore, it may not be accurate to conjecture that any face mask is beneficial to curb the viral spread. The type of mask fabric, its properties, and the number of layers also play a vital role in deciding the efficacy. It should also be noted that the smaller droplets might bypass a single layer mask where pore size is ∼100 *μ*m. Mask filtration efficiencies to such smaller sized (<100 mm) droplets have been extensively studied in the existing literature.^20,42–47^ However, in this work, we essentially demonstrate an additional route in which a large-sized droplet impingement with a single- or multi-layer mask can atomize into smaller droplets/aerosols. This suggests an additional mechanism of aerosol transportation that could be higher than what is predicted by considering mask filtration efficiencies alone.

## EXPERIMENTAL METHODOLOGY

Droplet impact dynamics onto the mask (both new and washed) were studied using high speed shadowgraphy imaging. A micrometer-sized DI water droplet (d ∼ 550 *μ*m diameter) was generated at 2 to 10 m/s impinging velocities using a pressurized liquid and piezoelectric actuation-based droplet dispenser (Nordson pico-pulse). In this dispenser, the velocity and size of the ejected droplet can be varied by changing the liquid fluid pressure and valve opening time, respectively. Still, some variation in droplet sizes is observed at different velocities, which are shown in Table I. It must be noted that the droplet velocity and size vary depending on the specific event, namely, normal breathing, coughing, sneezing. However, this variation is only addressed in the present study by changing the droplet impact velocity (∼2–10 m/s) and keeping a fixed droplet diameter (approximately). This choice is made as droplet penetration through a mask layer primarily depends on impact velocity, pore size and is independent of initial droplet diameter (will be shown later), which has been verified in the previous literature.^28,32,48^ Furthermore, the focus was kept on identifying the effectiveness of different mask fabrics (resulting in different pore sizes); therefore, parametric analysis based on different droplet sizes is not analyzed.

**TABLE I.** The variation in the experimental values of droplet diameter, velocity, % volume penetrated, and aerosol generated.

Velocity (m/s)	Diameter (*μ*m)	Error in % penetrated volume (%)	Error in % penetrated aerosol volume (%)
2.044 ± 0.01	571.372 ± 8.79	1.70	⋯
3.769 ± 0.02	510.039 ± 10.21	4.10	0.41
5.961 ± 0.08	449.622 ± 9.03	4.62	7.39
7.925 ± 0.08	516.572 ± 11.65	5.23	7.42
10.324 ± 0.10	544.108 ± 8.56	10.65	11.85

### High-speed shadowgraphy

**FIG. 2.**
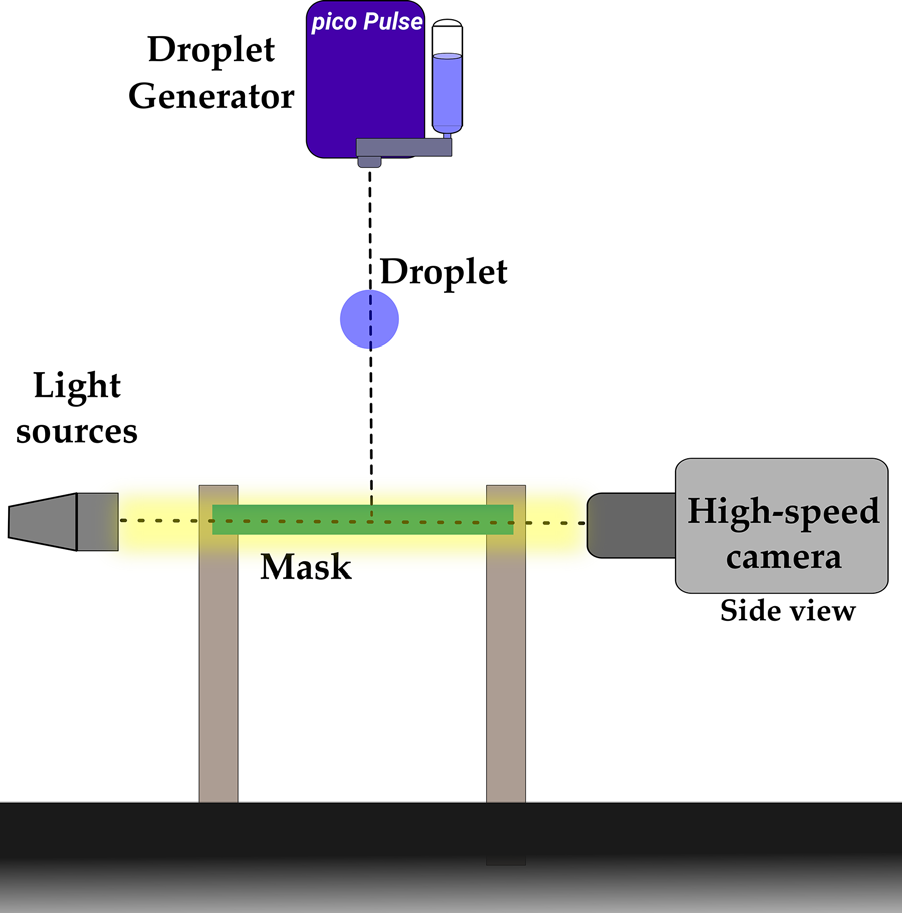
The experimental setup used for the investigation: two high-speed cameras were used to capture the side and top view of the droplet impingement on the cotton fabric surface. A high intense light source was used to capture the high-speed dynamics of droplet impact.

Two high-speed cameras (Photron SA5) coupled with TOKINA MACRO 100 F2.8 D with 2 × 36 mm^2^ extension tubes for side view imaging were focused on the droplet's plane of descent (see Fig. 2). Veritas (Constellation 120E, 120W) light source was used to study penetration dynamics over fabric surface. The camera acquisition rate was kept at 20 000 frames per second for low-speed droplet impingement (*V* ∼ 2–6 m/s) and for high-speed (*V* ∼ 8 and 10 m/s), and 30 000 frames per second were used as shown in Fig. 2. The penetrated droplet sizes are determined from shadowgraphy images. The captured images are binarized using intensity thresholding. From this area, the equivalent diameter is calculated to estimate the volume of each droplet. The volume summation of all such daughter droplets after impingement is done to determine the % volume penetration. The measurements were only done for droplets emerging from penetrated ligaments (after ligaments breakup into discrete daughter droplets). The obtained daughter droplet diameter of at least 20 experimental runs is then used to make the probability distribution of atomized droplets. Furthermore, for the penetrated volume quantification, the out of focus droplets (from out of focus ligaments) are assumed to contribute the same amount of volume as contributed by droplets emerging from the in-focus ligament. We have, however, minimized this error by averaging over large number of experimental visualizations from multiple azimuthal angles. The visualizations exhibit that the ligaments emerging from the pores are all of similar thickness for a given mask sample. This further ensures lower error count in volume estimation. The volume of aerosol generated is calculated based on the number of droplets below 100 *μ*m. A scale bar has been added to all the images for displaying the spatial resolution.

Image processing on the raw data was performed for edge detection to estimate the droplet size and velocity using MATLAB 2020b and ImageJ software. We used python3.7 using Anaconda environment for plotting. The movies were made using Kdenlive software. Experimental methodology is similar to Sharma *et al.*^32^

## MATERIAL SELECTION AND CHARACTERIZATION

**FIG. 3.**
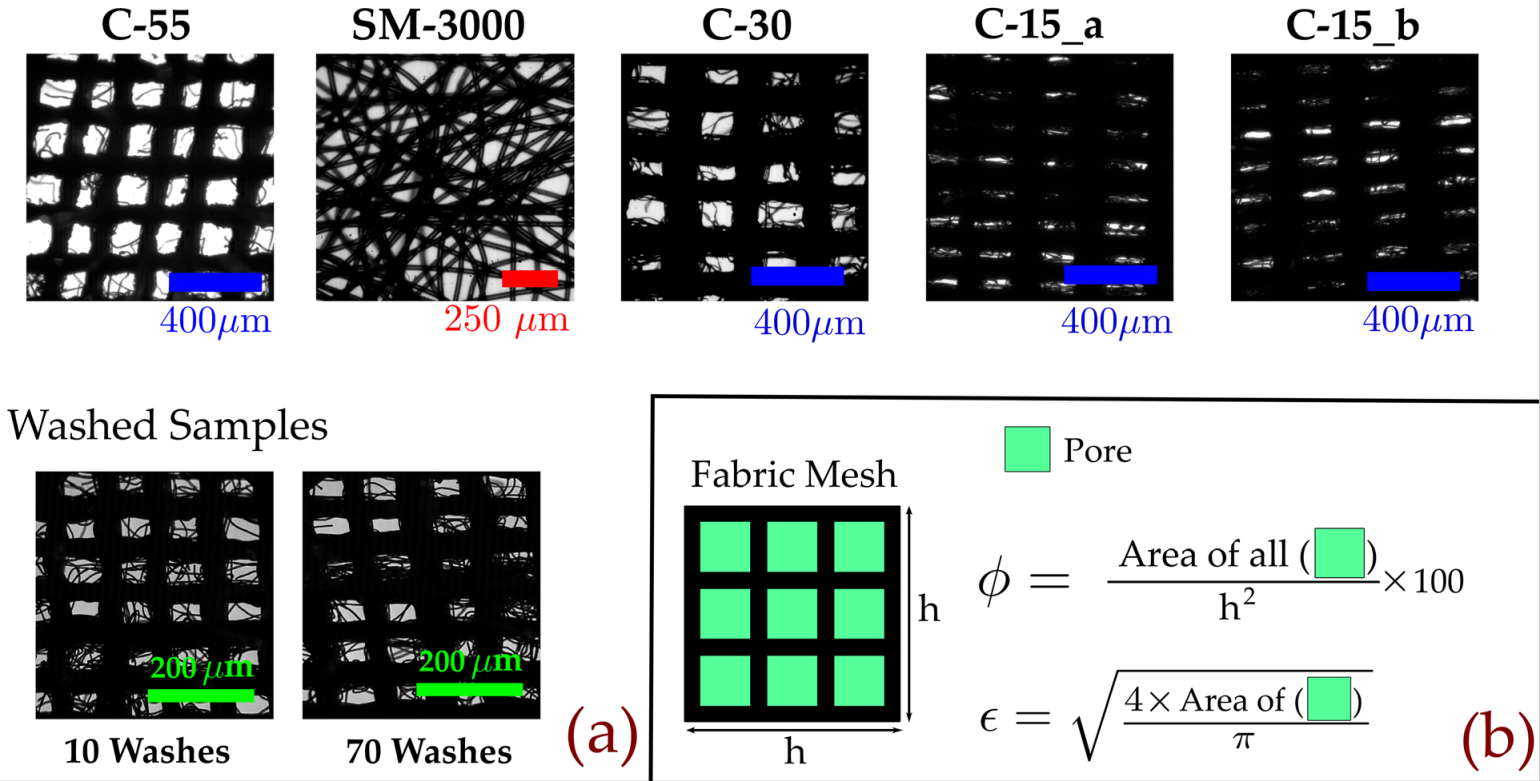
Samples and their characteristics: a magnified images of the samples, which were used in the experiments, are shown above. (a) Porous network of different cotton, surgical masks, and washed masks used in this work. (b) Methodology for obtaining pore size (φ) and porosity (ϵ).

In this study, different commonly available realistic cotton fabrics with different porosity and pore sizes were procured from the local market and characterized; properties and their respective contact angles are tabulated in Table II, and the magnified images of the samples are shown in Fig. 3. These specific cotton-fabric materials were selected based on their daily usage and the propensity of people to cover their face using these cloth materials (their common names are also mentioned in Table II). The contact-angle (*θ*) mentioned in Table II is based on the initial value measured just after the droplet has been dispensed on the fabric. Shadowgraphy experiments were performed for the measurement of contact angle. A fixed volume (6 *μ*l) of droplet using a micro-pipet (least count 0.5 *μ*l) was kept on the fabric surface (see the supplementary material, Fig. S3).

**TABLE II.** Characteristics of different cotton fabric and surgical masks used for the study: porosity and pore size of the samples have been tabulated. [C and SM represents cotton-fabric and surgical-mask respectively, the numbers represent the parameter η (number density of the pores) for a given sample.]

S. No.	Samples	Porosity ϕ (%)	Pore size *ϵ* (*μ*m)	Contact-angle *θ* (°)	*η* = *ϕ/ϵ*^2^ (*μ*m^−2^)	Thickness *t_m_* (*μ*m)
1	C-55 (summer stole)	25.037 ± 1.38	213.281 ± 6.84	65.45 ± 1.43	5.50 × 10^–6^	254.228 ± 14.49
2	SM-3000 (surgical mask)	20.09 ± 1.56	24.525 ± 2.95	130 ± 3.55	3.34 × 10^–4^	300 ± 18.15
3	C-30 (handkerchief)	9.302 ± 2.01	179.214 ± 4.40	67.37 ± 2.29	2.90 × 10^–6^	196.8 ± 10.41
4	C-15_a (coarse cotton towel)	2.237 ± 0.03	119.376 ± 6.88	43.8 ± 1.11	1.57 × 10^–6^	332.526 ± 11.09
5	C-15_b (cotton towel)	2.117 ± 0.26	117.964 ± 6.80	41.3 ± 1.89	1.52 × 10^–6^	340.908 ± 13.19

Side view images were captured, and contact angles for all the samples were measured using the ImageJ software using the angle measurement tool. The methodology used for obtaining effective pore size is shown in Fig. 3(b). The green colored area of all samples is obtained using image processing. This involves binarization of raw image and determining pore area. The effective pore size (*ϵ*) is determined from pore area ($A_{\mathrm{pore}}$) using $\epsilon =\sqrt{\left(\frac{4}{\pi }A_{\mathrm{pore}}\right)}$. Average pore size is used as representative value for that mask. Same procedure is used for all masks.

A detailed experimental investigation has been conducted to mimic the individual respiratory droplets ejected from an infected person onto the inner side of the mask (see Figs. 1 and 2). For DI water (σ=72 mN/m), the surface tension was found to be similar to that of cough droplets (σ=65.9 mN/m).^32,49^ However, the other fluid properties between the model cough droplet used in the present work and the actual cough droplet could be different. The important fluid properties relevant to droplet penetration are surface tension, viscosity, and viscoelasticity. As noted by Vontas *et al.* (2020),^48^ at high impact velocities, the significance of droplet fluid properties on penetration becomes less critical. Nevertheless, in our earlier work,^32^ we have performed additional experiments with surrogate respiratory liquid [0.9% by weight NaCl, 0.3% by weight gastric mucin, and 0.05% by weight Di-Palmitoyl-Phosphatidyl-Choline (DPPC)].^50^ The critical penetration characteristics do not show any discernible difference,^32^ suggesting a negligible influence of droplet fluid properties at high impact velocities. However, the fluid in the throat of a sick person could be different from common saliva; thereby, slight discrepancies in the results can be expected.

## RESULTS

### Droplet penetration criteria

Two penetration criteria based on viscous dissipation (based on Reynolds number ${Re}_{\epsilon }$) and surface tension (based on Weber number ${We}_{\epsilon }$) are considered in this work, which are essential for droplet penetration to occur.

Sahu *et al.*^28^ reported that for any fiber–liquid combination, there exists a threshold impact velocity above which liquid can penetrate the porous network of fibers irrespective of its hydrophobicity and initial mass. For the hydrophobic fabric of pore size (ϵ) and thickness (*t_m_*), the liquid ligaments can pass through the porous network if its impact kinetic energy $\left[E_{k}∼\rho d^{3}\;{\left(\frac{Vd}{\epsilon }\right)}^{2}\right]$ exceeds the viscous dissipation $\left[E_{d}∼\frac{\mu }{\epsilon }(\frac{Vd}{\epsilon })d\epsilon {\left(\frac{d}{\epsilon }\right)}^{3}\;{t_{m}}^{}\right]$. This offers the criterion that droplet penetration occurs for the condition:^28,32^ ${Re}_{\epsilon }\left(\frac{\epsilon }{t_{m}}\right)\gg 1$. The fabric of the mask would absorb some liquid (as the material itself is porous), which might result in additional viscous dissipation. However, such effects are not taken into consideration in the present work.

However, from Table II, except for SM-3000 (surgical mask) at 2 m/s, it is observed that the value of the above-mentioned parameter is ≫O(1). This is because viscous dissipation is negligible in all other samples due to the comparatively larger pore size (>100 *μ*m). This indicates that penetration should always occur for all impingement velocities, which is not in full agreement with experiments (see Table III). This implies that using only viscous dissipation to determine the penetration criteria is not adequate.

**TABLE III.** Penetration criteria for different impingement velocities and samples based on viscous dissipation: The ${Re}_{\epsilon }\left(\frac{\epsilon }{t_{m}}\right)$ is calculated for each sample for different velocities. The value of the parameter should be ${Re}_{\epsilon }\left(\frac{\epsilon }{t_{m}}\right)1$ for penetration to occur. [For lower values of ϵ(SM-3000), the criterion is more effective]. The red color font indicates that this criterion is inadequate to predict the penetration for respective samples. Experimentally, green color fill indicates that penetration does not occur, and orange color fill indicates that penetration occurs.

Sample	$\frac{t_{m}}{\epsilon }$	${Re}_{\epsilon }\left(\frac{\epsilon }{t_{m}}\right)$				
		2 m/s	4 m/s	6 m/s	8 m/s	10 m/s
SM-3000	12.23	4.491809	8.983617	13.47543	17.96723	22.45904
C-15_a	2.86	93.65292	187.3058	280.9588	374.6117	468.2646
C-15_b	2.82	93.75573	187.5115	281.2672	375.0229	468.7787
C-30	1.10	365.6312	361.7163	542.5744	723.4325	904.2907
C-55	1.19	400.8717	801.7434	1202.615	1603.487	2004.359

Hence, an additional criterion based on droplet surface tension is derived in this work, which suggests that the liquid droplet can penetrate through the fabric only when the resisting capillary pressure inside the fabric pores (∼4*σ*/ϵ) exceeds the impact dynamic pressure (∼*ρV*^2^) that the droplet exerts on the fabric.^51^ Hence, the droplet can only penetrate a porous surface or mesh when the dynamic pressure of the droplet is high enough to overcome the capillary pressure of the pores, as shown in the following equations:

$$\rho V^{2}>\frac{4\sigma }{\epsilon },$$ (1)

$$\frac{\rho V^{2}\epsilon }{4\sigma }>1,$$ (2)

$${We}_{\epsilon }>1,$$ (3)

where *ρ* is liquid density, *σ* is the surface tension between the water–air interface, *V* is impact velocity, *ϵ* is pore size, and *We* is the weber number. The calculated values of ${We}_{\epsilon }$ are tabulated in Table II.

From Table III, for three samples C-15_a, C-15_b, and SM-3000 at 2 m/s, the criterion shows that ${We}_{\epsilon }<1\;or\;{We}_{\epsilon }∼1$. For all other cases at higher velocities, the criterion shows ${We}_{\epsilon }>1$ for all the samples. From this, we can infer that this criterion is in good agreement with experiments, suggesting that a higher value of ${We}_{\epsilon }$ leads to penetration.

The viscous dissipation criterion ${Re}_{\epsilon }\left(\frac{\epsilon }{t_{m}}\right)\gg 1$ has indicated that penetration will occur in all other cases except for sample SM-3000 at 2 m/s. However, the ${We}_{\epsilon }$ criterion indicates that except for three samples C-15_a, C-15_b, and SM-3000 at 2 m/s, penetration is possible in all other samples. Hence, by using two-part penetration criteria, it can be predicted that the droplet should not penetrate the fabric in the three samples C-15_a, C-15_b, and SM-3000 at 2 m/s (see Table IV).

**TABLE IV.** Penetration criteria for different impingement velocities and samples based on capillary forces: ${We}_{\epsilon }$ is calculated for each sample for different velocities. The value of the parameter should be ${We}_{\epsilon }>1$ for penetration to occur. Experimentally, green color fill indicates that penetration does not occur, and orange color fill indicates that penetration occurs.

Sample	${We}_{\epsilon }$				
	2 m/s	4 m/s	6 m/s	8 m/s	10 m/s
SM-3000	0.354	1.217	30.472	49.306	79.835
C-15_a	1.559	6.119	155.266	255.737	436.506
C-15_b	1.45	6.018	146.635	247.44	432.001
C-30	2.534	8.95	217.132	375.628	639.887
C-55	2.974	11.268	261.266	473.358	763.825

Experiments are performed with different droplet diameters ranging from 250 to 650 *μ*m for various cloth fabrics. It was found that two-part penetration criteria are independent of the droplet size as long as d>ϵ.^28^ It is to be noted that specifically for cotton fabric samples used in the current study, the viscous dissipation criterion does not contribute much in the determination of penetration due to larger pore size [$O\left(\epsilon \right)∼O(d)]$; however, it is effective in estimating the penetration criteria for the SM-3000 sample, which has a lower pore size [$O\left(\epsilon \right)<O(d)]$ of 20–30 *μ*m.

Hence, it can be inferred that at ${Re}_{\epsilon }>O\left(10^{2}\right),$ the viscous dissipation-based criterion is not effective in giving an accurate penetration prediction. However, there can be an intermediate-range of ${Re}_{\epsilon }$ where both the viscous dissipation and the capillary effects can significantly contribute toward penetration criteria. It is also to be noted that ϵ affects both surface tension and viscous dissipation while $t_{m}$ only influences viscous dissipation. The effect of ${\;\epsilon ,\;t}_{m}$ on viscous dissipation can be understood in a better fashion by their relative contributions to viscous dissipation. Lower is the value of ϵ, higher is the velocity gradient in the pore leading to higher viscous dissipation. Similarly, for a higher value of $t_{m}$, more work needs to be done by the liquid to pass through the long length of the pore. Hence, the ratio $\frac{t_{m}}{\epsilon }$ can be used to ascertain the relative importance of viscous dissipation and capillary force, as shown in Table II. The ratio $\frac{t_{m}}{\epsilon }$ is found to be of the order O(1) or less when the surface tension effects are dominant; however, the value of $\frac{t_{m}}{\epsilon }$ is found to be of the order $O(10^{2})$ or more when viscous dissipation is dominant. It is clear from Table III that the value of $\frac{t_{m}}{\epsilon }$ for SM-3000 is 12.23, which is clearly an order higher than all the other samples. Also for SM-3000 at 2 m/s, the value of ${Re}_{\epsilon }\;∼55$, which is at least an order less than all the other samples at all velocities. Both these arguments are sufficient to infer that at 2 m/s, the viscous dissipation criterion is effective only in the case of SM-3000 or similar masks with low pore size.

In the case of a multi-layered mask, since the pores in adjoining layers will not be perfectly aligned, the effective pore size (ϵ) and porosity (φ) decreases. This decrease in ϵ will result in an increase in capillary pressure and viscous dissipation, leading to a lesser propensity of penetration with each additional layer. It is to be noted that the propensity of an impacting droplet to penetrate through the mask layer depends only on the fabric pore size. Thus, for penetration to occur, the porosity of fabric is not relevant. However, the extent of droplet volume penetrated/aerosolized through the fabric depends on the number of pores under the impacting area; thereby, it depends on the mask's porosity.

### Droplet impingement on a porous surface

It is observed that three different phenomena emerge because of droplet impingement on a single-layered porous fabric.

**FIG. 4.**
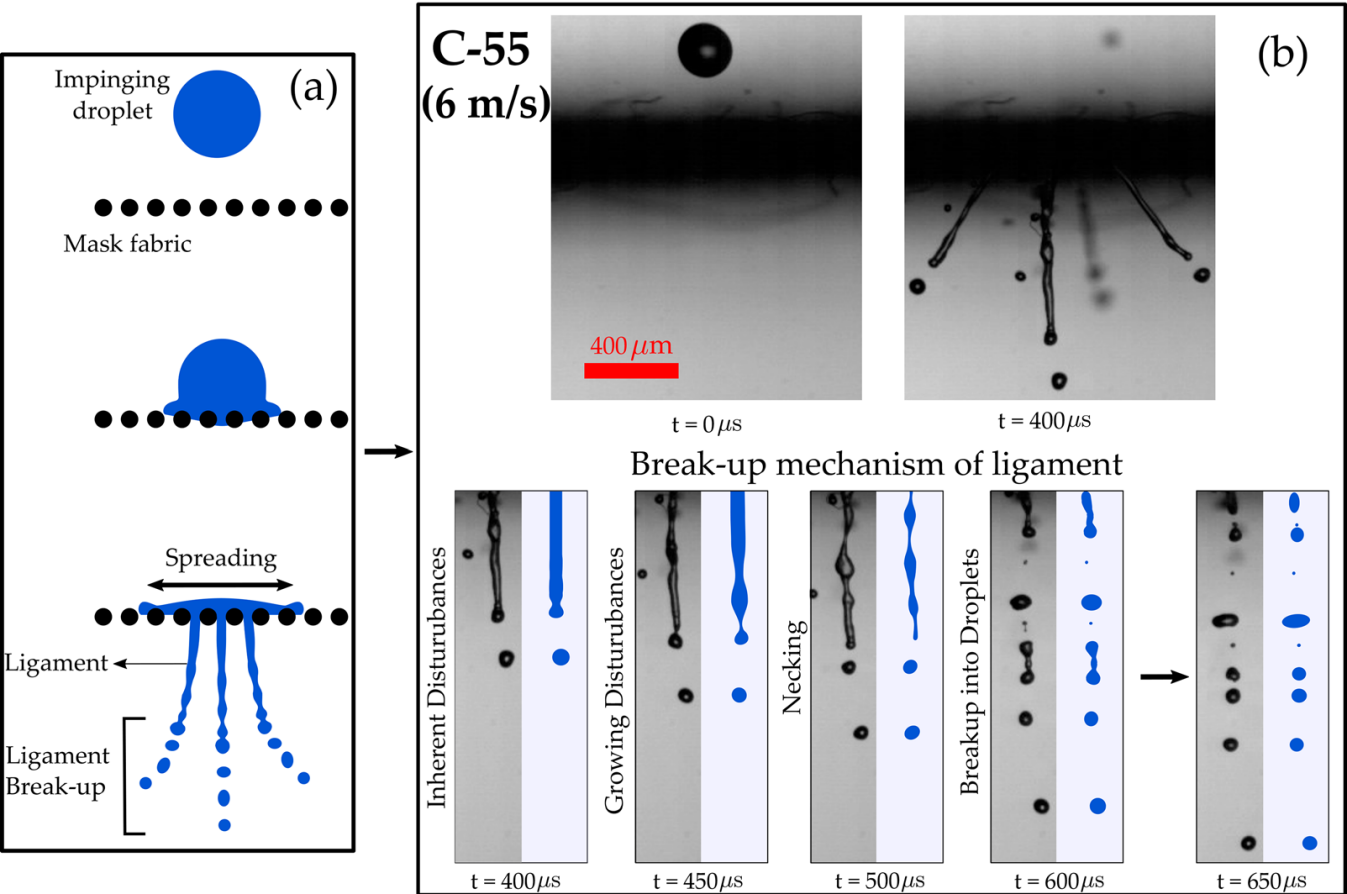
Droplet spreading and penetration on cotton fabric during impact: (a) schematic of the droplet impingement on a single layer fabric showing spreading and penetration of the droplet; (b) time series of the ligament breakup has been shown at 6 m/s for C-55. We can observe disturbances in the form of a wave that grows with time leading to necking and breakup due to Rayleigh–Plateau instability. Multimedia view: https://doi.org/10.1063/5.0061007.1
10.1063/5.0061007.1

One is the penetration of the model cough-droplets through the pores, the other is spreading of the droplet on the surface of the fabric and the retention of the remaining volume by the fabric [see Fig. 4 (Multimedia view)]. The volume of the droplet that gets blocked by the fabric spreads radially along the fabric surface. A detailed study of droplet penetration mechanism and effect of fabric properties is provided in this work.

### Overview of the effect of fabric properties on droplet penetration

**FIG. 5.**
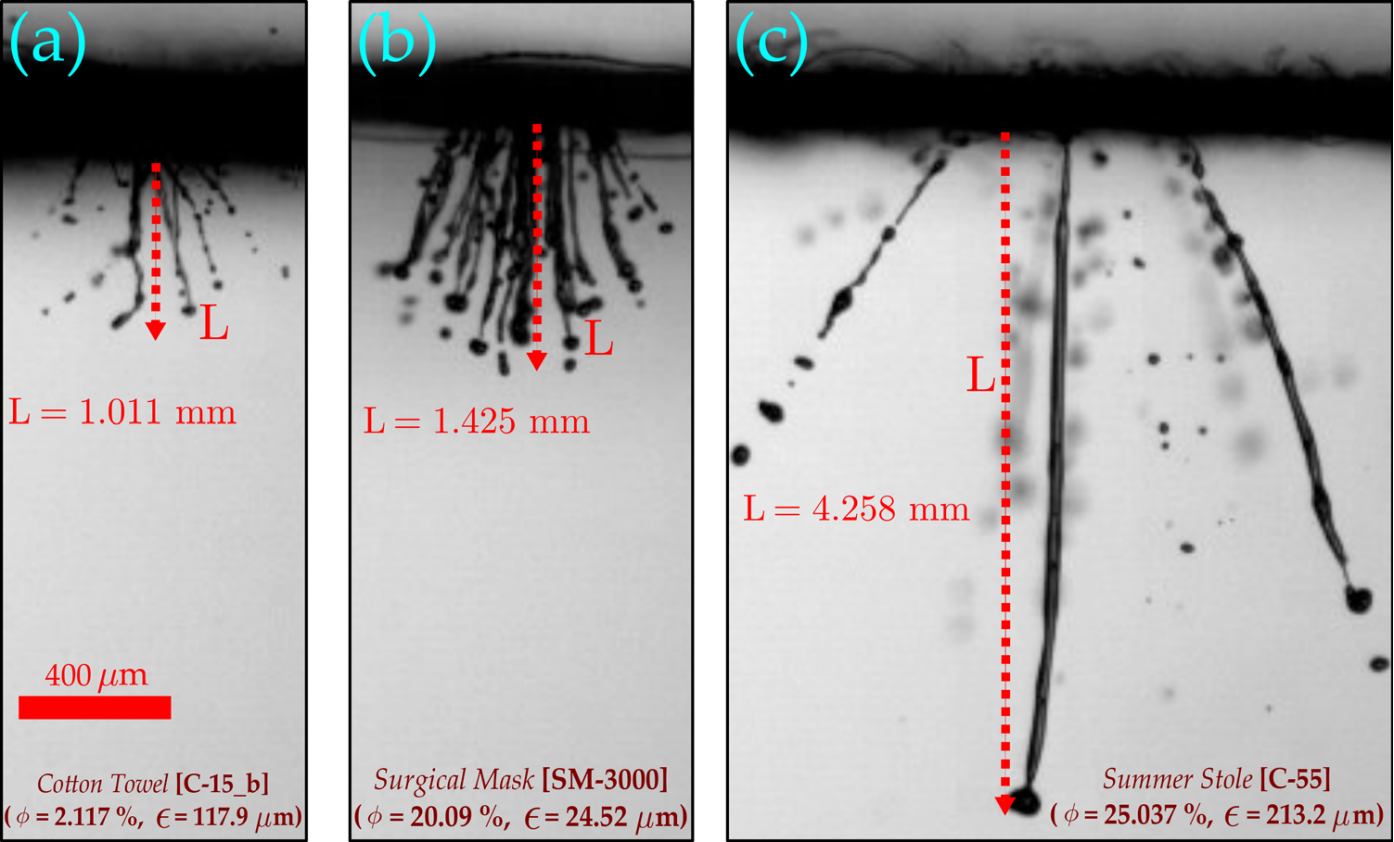
The variation of ligament length for different samples: The length of the largest ligament during penetration is shown above for different samples at 10 m/s. The ligament length is the smallest for cotton towel followed by surgical mask and summer stole. With an increase in porosity, the %volume penetration increases as the more open area is available to pass through the mask. (a) The ligament length measured for cotton cloth is shown for 10 m/s impingement; the length depends on the velocity and pore size. Compared to summer stole (see c), cotton towel has smaller pores, which result in smaller size ligaments. The ligaments break into very tiny daughter droplets, which may potentially result in aerosols. (b) Despite the smallest pore size, the ligament size is larger for the surgical mask compared to cotton towel. This is because of the coalescence of the ligaments. Multimedia view: https://doi.org/10.1063/5.0061007.2
10.1063/5.0061007.2

The percentage volume of the penetrated liquid is a global quantity that describes the net cumulative volume of liquid that passes through all the pores of a fabric. Hence, it depends on both porosity (φ) (a global property) and pore size (a local property), and it also increases with the impingement velocity (cough intensity). The porosity is the percentage area available on the fabric; more liquid passes through the fabric with an increase in porosity. However, smaller pore size (ϵ) means larger capillary pressure [Eq. (1)] and higher viscous dissipation,^28^ each of which may result in the formation of the shorter ligament on penetration. This also explains the formation of longer ligaments in larger pore-sized masks [see Fig. 5 (Multimedia view)].

A mask with high porosity and smaller pore size implies an increased number of smaller pores, which means that many small-sized ligaments are formed in this case (see Figs. 4 and 5). However, for masks with low porosity and larger pore size, fewer ligaments are formed. The ligaments formed in this case are longer (see Fig. 5). These longer ligaments breakup into many daughter droplets (see Figs. 4 and 5). The ligament breakup mechanism will be discussed in ligament breakup section.

### Ligament breakup

The volume of model cough-droplets passing through the pores of the fabric extrudes out in the form of ligaments and breaks up further into smaller droplets due to the Rayleigh–Plateau instability. The inherent disturbances present in the liquid stream passing through the pores may grow or decay with time at different rates, depending on the wavenumber and ligament size. Growth of the natural disturbances will cause necking and eventual breakup of the ligament into tiny droplets (see Fig. 4).

A part of the initial kinetic energy of the impinging droplet is lost to the viscous dissipation while passing through the fabric pores and the associated capillary pressure. The other portion enables the spreading of the liquid along the fiber surface. The remaining available energy is converted into kinetic energy of ligaments with the extrusion of the liquid through the pores. The ligament finally breaks up into daughter drops due to Rayleigh–Plateau instability.

### The combined effect of pore size (ϵ) and porosity (φ) on volume penetration and aerosol generation

As discussed above, the penetrated volume of droplets depends on the impingement velocity, porosity, and pore size of the fabric. A pore number density (*η*) parameter has been formulated (to combine both the fabric properties; porosity and pore size), which is defined as

$$\eta =\varphi /\epsilon^{2}.$$ (4)

**FIG. 6.**
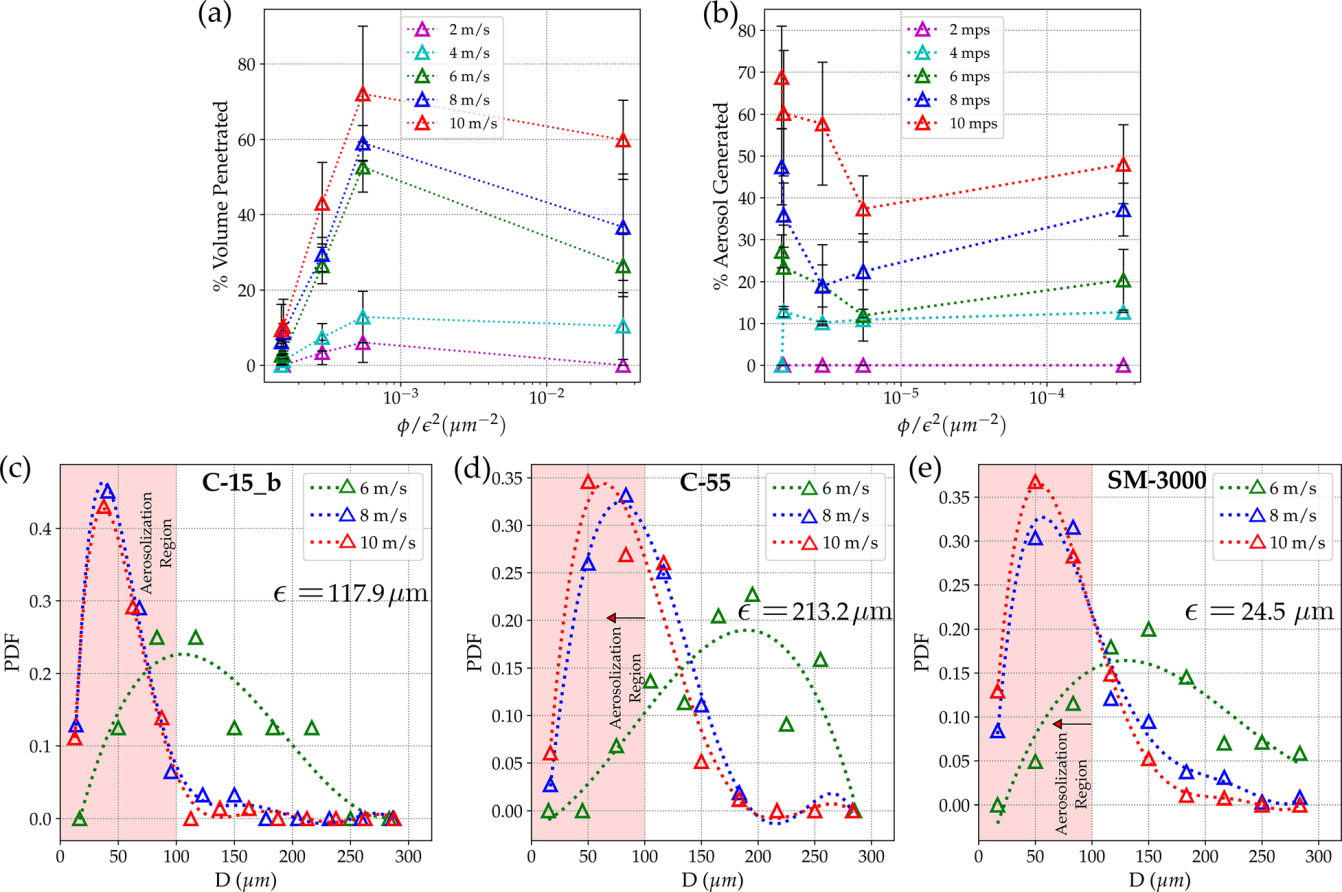
Percentage volume penetrated through the cotton mask and aerosolization range for different cotton fabrics at different velocities: (a) the percentage volume penetrated plotted against the number density of the pores (*η* = *ϕ/*$\epsilon^{2}$), *ϕ* is the porosity, and ϵ is the pore size for the single-layer of a given sample. (b) Percentage of the penetrated droplets in aerosolization range (≤100 *μ*m). (c)–(e) Droplet size distribution plotted as the probability density function of the penetrated droplets at different velocities for cotton towel (C-15_b), summer stole (C-55), and surgical-mask (SM-3000), respectively [(c)–(e), in an increasing order of *η*].

This is a unifying quantity that includes the combined effect of pore size and porosity of the fabric. It is the effective pore number density, η∼ (available liquid flow area/total area)/(area of each pore), i.e., the number of pores available per unit area for the liquid droplet to pass through in a given fabric. Intuitively, more space available results in more penetration/chance of penetration. The local effect of pore size and pore density is normalized using this parameter that effectively reduces the multifaceted effects into one single parameter that is physically intuitive. It is clear from Fig. 6(a) that the %volume penetrated increases with an increase in velocity. However, extreme values of *η* will lead to a lower % of volume penetration. For very low *η* (porosity is very low and pore size is very high), the effective area available for penetration is significantly less and, hence, leads to lower % volume penetration. Moreover, for the large value of *η*, there are high numbers of smaller pores available for penetration. These small pores provide larger resistance for droplets to penetrate due to the larger capillary forces and viscous dissipation, restricting the amount of penetrated volume percentage. The optimum values for porosity and pore size are needed to achieve a higher % of volume penetration near the intermediate values of *η* as shown in Fig. 6(a). (Value of *η* for each sample is tabulated in Table I.)

The size distribution of droplets (post-penetration) for different single-layer fabric samples has been studied at different impingement velocities. It follows a bell-shaped trend where the maximum peak value increase and shifts toward smaller diameter sizes (left) as the impingement velocity (cough intensity) are increased, as shown in Figs. 6(c)–6(e); this implies that the probability of breakup of ligament into smaller droplets increases with higher impingement velocities. However, at lower velocities, the probability of forming larger-sized droplets is higher (see Fig. 4). This suggests that, at higher impingement velocities, more secondary atomization and aerosolization are observed (see Fig. 5). With the increase in impingement velocity, the ligaments stretch and become longer and thinner (see the supplementary material, Fig. S2), resulting in the formation of larger number of smaller daughter droplets. For 6 m/s, the total penetrated volume is contributed by the lesser number of larger daughter droplets. However, in the case of higher velocity i.e., 8 m/s, the total penetrated volume constitutes the summation of volume contributed by larger number of small daughter droplets.

The probability density function (PDF) of droplet size is plotted for three samples having the lowest, medium, and highest *η* values in Figs. 6(c)–6(e), respectively. It is to be noted that very few droplets (<8) are formed at impingement velocities of 2 and 4 m/s. Hence, PDFs have been plotted for only 6, 8, 10 m/s. From Figs. 6(c)–6(e), it can be observed that for a given velocity, the maximum peak is observed to initially shift toward the right and then to the left, as *η* is increased (for samples C-15_b, C-55, and SM-3000, respectively). The increasing and decreasing trend in the most probable daughter droplet diameter can be explained by the increase in the pore size (ϵ) from C-15_b (∼100 *μ*m) to C-55 (∼200 *μ*m) and a decrease in the pore size from C-55 (∼200 *μ*m) to SM-3000 (∼20 *μ*m). Larger pore size leads to the increased thickness of ligaments, resulting in the formation of larger-sized droplets, i.e., away from the aerosol range (see Fig. 5 (Multimedia view) and supplementary material). However, if we compare the PDF of the two samples [see Figs. 6(c) and 6(e)], the droplet size corresponding to the maximum probability is higher in the case of SM-3000 than C-15_b despite the smaller pore size of SM-3000. This can be attributed to the fiber mesh pattern of SM-3000 [see Fig. 3(a)], where the distance between the pores is very less due to the reduced fiber thickness (single layer), which might cause the coalescence of the ligaments emerging from adjacent pores. Coalescence can result in relatively larger droplets than expected for the corresponding pore size (see Fig. 5 (Multimedia view) and the supplementary material).

There is an increase in the percentage of penetrated droplets in the aerosolization range (≤100 *μ*m) [see Fig. 6(b)] with an increase in *η.* This is why cloth masks made of C-15_b have the highest droplet filtration efficiency among the fabrics. However, Fig. 6(a) shows that any single-layer fabric will not be beneficial to curb the spread of COVID19 as it leads to varying levels of aerosolization. The %volume penetration trend shown in Fig. 6(a) matches with the pdf of droplet size distribution in Figs. 6(c)–6(e), where the droplet size corresponding to the maximum probability is highest in the case of C-55 [see Fig. 6(d)]. It is to be noted that minimizing the percentage volume penetration is the primary concern of any mask to mitigate the extent of disease spreading. In Figs. 6(c)–6(e), the droplet size distribution for 6and 8 m/s differs significantly due to higher stretching of ligaments at higher velocity resulting in smaller droplet sizes compared to 6 m/s, while that the data nearly overlap for 8 and 10 m/s, which is discussed above in this section. Similar results were also reported by Sharma *et al.*^32^ and Vontas *et al.*,^48^ where at high impact velocity (i.e., high Weber number and Reynolds number), droplet shows similar penetration volume/distribution/characteristics and variation was only observed at lower impact velocities.

It is interesting to note that with an increase in porosity for a given pore size, a more significant number of ligaments are formed as more pores are available for the cough-droplets to pass through, i.e., a larger number of droplets are formed with the length and thickness of each ligament affecting the droplet size distribution. Thus, the larger porosity of the sample leads to a higher percentage of penetration volume [see Figs. 5 and 6(a)]. For two fabric samples (SM-3000 and C-55), both of higher porosity but different pore sizes (ϵ = 24.525 *μ*m and ϵ = 213.281 *μ*m), it is observed that in the case of smaller pore size, shorter ligaments and a larger number of smaller droplets are formed. Contrarily, longer ligaments and a larger number of bigger droplets are formed in the case of larger pore size. (The effect of ϵ  and  φ is shown in Figs. 5 and 6). The data presented above pertain to a single layer. However, % volume penetration of model cough-droplets was checked for multi-layers as well, and a significant reduction in the % volume penetration was observed with three-layer masks for all the samples.

### Effect of washing

**FIG. 7.**
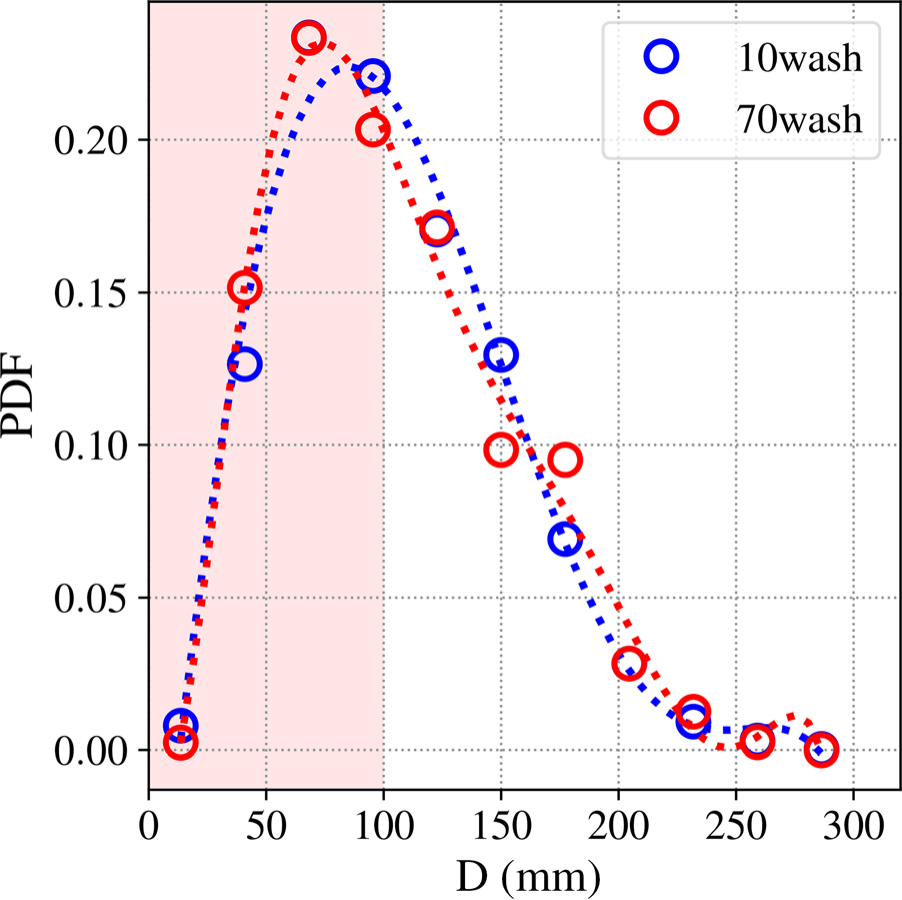
The PDF of droplet distribution for washed cotton masks: the probability density function for washed samples shown in the figure at 10 m/s.

The effect of the number of wash cycles imposed on a cloth sample has been investigated for mask efficacy. Masks were hand washed using commercial detergent using water at room temperature. In the present study, no significant difference is observed with the number of washes (see Table V), which can be reflected in the similar fabric properties of the samples subjected to either ten wash cycles or 70 wash cycles. The droplet size distribution is plotted in Fig. 7 as a probability distribution function, and a bell-curve-like trend is also observed for washed samples, which peaks at a droplet size of around ∼80 *μ*m.

**TABLE V.** Properties of washed cotton masks: the cotton mask was washed for 10 and 70 times, and their properties are shown below at 10 m/s.

	10 Washes	70 Washes
Porosity (%)	16.904 ± 2.36	20.344 ± 3.19
Pore size(*μ*m)	171.023 ± 51.99	178.008 ± 9.69
*η* = Porosity/poresize^2^ (*μ*m^−2^)	5.8 × 10^–6^	6.4 × 10^–6^
%Volume penetrated @ 2 m/s	3.103 ± 0.44	2.295 ± 1.07
%Volume penetrated @ 10 m/s	47.9584 ± 16.09	49.934 ± 8.99
%Aerosol	11.989 ± 3.16	10.054 ± 3.67

## CONCLUSIONS

The penetration phenomenon is not dependent on just one parameter like surface tension or viscous dissipation. Both these effects are necessary to be considered to determine the possibility of penetration. The viscous dissipation criterion is effective only when the pore size is smaller [<O (100)]. Thus, both the viscous dissipation and ${We}_{\epsilon }$ criteria are necessary for an effective prediction of penetration for any given sample at any given impingement velocity. These criteria suggest that except for the three samples C-15_a, C-15_b, and SM-3000 at 2 m/s, penetration is possible for all other samples for single-layer, which is in good agreement with experiments. With an increase in the number of layers, effective pore size (ϵ) decreases, which suggests that penetration tendency will decrease with each additional layer according to the criterion.

The current study is directed toward those people who cannot use the recommended N95 mask due to lack of convenience, availability, or economic and demographic reasons. This study recommends the safer option among commonly available fabrics that the said population generally uses for homemade masks without compromising on actual safety against COVID-19. Experiments were primarily performed with single-layer fabric, which showed significant %volume penetration (>25%) in all other fabrics except for C-15 samples, at higher impingement velocities (>4 m/s) (well correlated with cough intensity). We checked for the penetration with the least-safe C-55 sample in its three-layered configuration, which showed minimal penetration. Hence, through this study, based on the low %volume penetration (<20% even at large impingement velocity ∼10 m/s), we recommend either the usage of coarse cotton towel or cotton towel (with at least three layers) as a face-covering if the person is unable to use N95 or surgical-mask. Three or more layered mask is recommended since it can significantly suppress aerosolization. However, for the unavailability of any such substitutes, one should always wear whatever makeshift mask is available.

Also, from this study, we advocate a parameter *η* along with the mechanism of atomization of droplets, which can be used to know the extent of efficacy of any given fabric used as a face mask material, as shown in Fig. 6(a). The droplet size distribution Figs. 6(b)–6(d) (which is essential for estimating the infection transmission) shows the presence of aerosol range droplets that are produced because of the atomization of larger droplets. In this paper, we have also shown the importance of multiple layers to minimize this aerosolization effect. Additionally, the effect of washing the mask has been investigated, which showed very little change in the fabric properties like pore size and porosity because penetration characteristics also remain similar. However, the effect of a higher number of wash cycles has not been investigated as part of the study.

## SUPPLEMENTARY MATERIAL

See the supplementary material for showing spreading of droplets on a mask fabric.

## AUTHORS' CONTRIBUTIONS

D.G. and G.V. contributed equally to this work.

## Data Availability

The data that support the findings of this study are available within the article and its supplementary material.
